# Profillin‐1 and Transgelin‐2: Actin Binding Proteins Expression in Early and Advanced Stages of Triple‐Negative Breast Cancer Receiving Neoadjuvant Chemotherapy

**DOI:** 10.1002/cnr2.70529

**Published:** 2026-03-30

**Authors:** Moazzam Ali Shahid, Shamim Mushtaq, Ayesha Khan, Uzma Naseeb, Ghulam Haider, Bushra Wasim

**Affiliations:** ^1^ Department of Biochemistry Ziauddin University and Hospital Karachi Pakistan; ^2^ Department of Biochemistry University of Karachi Karachi Pakistan; ^3^ Department of Biochemistry Jinnah Sindh Medical University Karachi Pakistan; ^4^ Department of Oncology Jinnah Postgraduate Medical Center Karachi Karachi Pakistan; ^5^ Department of Anatomy Ziauddin University and Hospital Karachi Pakistan

**Keywords:** ELISA, mass spectrometry, Profilin‐1, RT‐qPCR, transgelin‐2, triple negative breast cancer

## Abstract

**Background:**

Triple‐negative breast cancer (TNBC) is known for its more aggressive clinical behavior, poor prognosis, and distinctive patterns of metastasis. Neoadjuvant chemotherapy (NAC) can influence both tumor cells and the tumor microenvironment. Emerging evidence highlights the critical role of actin cytoskeletal dynamics in cancer progression.

**Aims:**

This study aimed to explore the expression of two actin binding proteins, profilin‐1 (PFN‐1) and transgelin‐2 (TAGLN‐2), before and after NAC. Their prognostic significance, particularly in response to NAC in TNBC, has not been fully elucidated. Their expression patterns may provide valuable insight into their prognostic value and treatment response.

**Methods and Results:**

This quasi‐experimental study included 54 females (aged 24–45 years) diagnosed with TNBC pre‐ and post‐treated NAC and age matched healthy donors (*n* = 30). The strategy encompasses sequential high‐resolution Reverse‐Phase Liquid Chromatography Mass Spectrometry (RPLC‐MS/MS) for identification of peptides followed by ELISA and RT‐qPCR. An *in silico* analysis, STRING and KEGG analysis were also employed. The PFN‐1 protein and gene expression in early stages (I & II) of pre‐NAC were significantly (*p* = 0.0001) higher. However, in the post‐NAC patients with advanced stages, expression was reduced. The TGLN‐2 protein concentration was significantly higher (*p* = 0.0001) in the pre‐NAC group at advanced stages compared with post‐NAC patients. Gene expression of TGLN‐2 is highly overexpressed in both groups at advanced stages. Moreover, tumor suppressor gene PTEN was downregulated at early stages of pre and post‐NAC groups and upregulated at advanced stages of post‐NAC. STRING and KEGG analysis showed that PFN‐1 and TGLN‐2 both displayed association with regulation of actin cytoskeleton.

**Conclusion:**

Aberrantly expression of actin binding proteins, PFN‐1 and TAGLN‐2 and tumor suppressor gene PTEN in pre and post‐NAC patients in different tumor stages compared with healthy individuals can provide valuable prognostic information and may have potential as a therapeutic target. The observed interactions of PFN‐1 with VASP and potential crosstalk with TAGLN‐2 point toward their collective role in actin cytoskeleton regulation and tumorigenesis.

## 1 Introduction

Triple‐negative breast cancer (TNBC) is a highly aggressive and rapidly growing tumor that is diagnosed at a later stage, associated with shorter overall survival and affects younger women [1, 2, 3]. Patients have higher recurrence and mortality rates following tumor resection compared with hormone receptor positive patients [4]. In patients with triple‐negative breast cancer, neoadjuvant chemotherapy (NAC) administered prior to surgery is commonly employed to improve surgical outcomes and assess treatment response, while adjuvant chemotherapy remains an option depending on disease stage and regional practice [5].

It has been established that oncogenic transformation of cells is often associated with significant alteration in the actin cytoskeleton, which plays a crucial role in cell shape, signaling, and motility. These changes in the actin cytoskeleton are not merely consequences of transformation but can actively contribute to cancer progression [6, 7].

Transglin‐2 (TAGLN‐2) and Profilins‐1 (PFN‐1) are large families of proteins present ubiquitously across all eukaryotic cells empowering actin cytoskeletal reorganization. Both have been shown to be obligatory molecular players for cell–cell interactions, cell motility, migration, proliferation, differentiation, and adhesion [8, 9, 10].

It is known that the increasing metastatic potential of tumor cells is strongly associated with the loss of actin filaments and a disorganized actin cytoskeleton. PFN‐1 plays a key role in multiple cellular pathways that regulate cell fate, extending beyond its classical function as an actin polymerization regulator. Changes in PFN‐1 expression levels significantly impact cell motility, proliferation, and apoptosis, making it a crucial player in cancer progression and other diseases [11]. An in vitro study showed that low PFN‐1 levels are associated with increased motility, invasion, and metastasis; however, in vivo, PFN‐1 depletion allows cancer cells to escape the primary tumor more efficiently, increasing metastatic spread [12].

TAGLN‐2 protein is highly expressed in tumor cells and plays a crucial role in cell morphology, transformation, and cancer progression. It is involved in various human malignancies, influencing tumor growth, invasion, and metastasis [13, 14, 15, 16]. However, it has a dual role in cancer, acting as a tumor suppressor and an oncogene; its loss has been associated with metastasis [17].

In breast cancer, pre and post‐NAC have been actively studied in the last decade. However, after NAC, tumors also show significant gene or protein expression changes that could be used to develop targeted therapies. Serum proteins that show differential expression across different stages of cancer could serve as valuable biomarkers for predicting and monitoring disease progression. Although PFN‐1 and TAGLN‐2 have been investigated in various aspects of cancer biology, their specific role in TNBC progression, particularly in patients receiving neoadjuvant chemotherapy (NAC), is not yet fully elucidated. Notably, there is a lack of evidence examining the dynamic expression changes of PFN‐1 and TAGLN‐2 at early and advanced stages, specifically through comparative assessment at pre‐NAC and post‐NAC time points, which may yield important insights into treatment response and disease progression.

## 2 Materials and Methods

### 2.1 Study Design, Duration and Setting

The study design selected for the present study was quasi experimental, *n* = 54 female (aged 24–45 years) diagnosed TNBC (defined TNBC as when IHC for ER, PR, and HER‐2 were negative) patients with and without metastases at Ziauddin Hospital and Jinnah Postgraduate Medical Centre (JPMC) between January 2022 and July 2023 were included. Patients included in our study received at least four cycles of NAC regimens with TNBC stages I–IV.

### 2.2 Sample Size Calculation

The sample size for this study comparing two proportions was calculated on https://select‐statistics.co.uk/calculators/sample‐size‐calculator‐two‐proportions/, by taking 21% and 49% proportion (TNBC consist of 20% of all breast cancers) at 95% confidence interval and 80% power and with 20% margin of error

$$\begin{array}{c} n=\left(\mathrm{Z\alpha}/2+\mathrm{Z\beta}\right)2\times \left(\text{p1}\left(1-p1\right)+\text{p2}\left(1-\text{p2}\right)\right)/\\ \;\left(p1-p2\right)2\;\text{With}\;20\%\text{Wastage}\;42+8=50 \end{array}$$

where Zα/2 is the critical value of the normal distribution at α/2 (e.g., for a confidence level of 95%, α is 0.05 and the critical value is 1.96), Zβ is the critical value of the normal distribution at β (e.g., for a power of 80%, β is 0.2 and the critical value is 0.84), and p1 and p2 are the expected sample proportions of the two groups.

### 2.3 Recruitment of Patients

**FIGURE 1:**
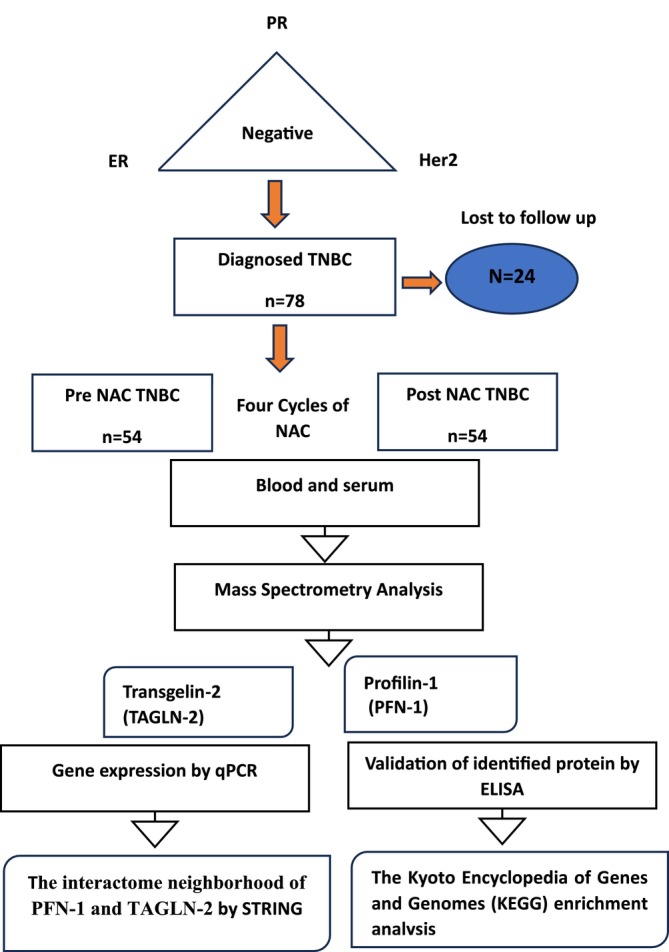
Workflow of the current study; female diagnosed Triple Negative breast Cancer (TNBC) were recruited when immuno‐histochemistry (IHC) for estrogen receptor (ER), progesterone receptor (PR), and human epidermal growth factor receptor (HER‐2) analysis were negative. Blood and serum were taken from TNBC (*n* = 54) patients with and without metastases. Mass spectrometry analysis has revealed two peptides PFN‐1 and TAGLN‐2 which was further validated by ELISA and mRNA expression was done by qPCR. STRING was used for protein–protein interaction (PPI) analysis and to explore biological association networks. Pathway analysis was performed on the selected proteins and genes PFN‐1 and TAGLN‐2 using KEGG analysis.

Blood samples were collected from patients prior to initiation of neoadjuvant chemotherapy (pre‐NAC) and after completion of four cycles of neoadjuvant chemotherapy (post‐NAC), before definitive surgical intervention. To determine the clinical tumor stages and nodal status, a standard bilateral mammography and ultrasound imaging of the breast were performed prior to the start of NAC. All research was performed in accordance with the Declaration of Helsinki and with relevant guidelines. Informed consent was obtained from all the participants and their legal guardians. Ethical approval for the study was obtained from the Research Ethics Committee (ERC) of Ziauddin University (Reference code: 1730120SMBIO). Figure 1 presents a summary of the workflow for the current study. A total of 54 diagnosed TNBC patients were included in the analysis after 24 patients were lost to follow‐up. Baseline characteristics of TNBC patients were shown in Table S1.

### 2.4 Mass Spectrometry

Patients (Pre and Post‐NAC) serum samples (10 μL) were supplemented with 10 μL of 8 M urea in 50mM Tris–HCL buffer, pH 8.5 and mixed well. Before sonication in a water bath for 5 min, 10 μL of 0.2% ProteaseMAX (Promega, USA) was added in 20% acetonitrile and 50mM Tris–HCL buffer. Additional 220 μL of 50 mM Tris–HCL was supplemented before determining the protein concentration by BCA assay (Pierce, USA).

Protein (25 μg) were reduced with 1,3 μL of 0.5 M dithiothreitol (ThermoFisher, Scientific, USA) in 50 mM Tris–HCL, incubated for 45 min at 37°C and alkylated with 3.2 μL of 0.5 M iodoacetamide (ThermoFisher, Scientific, USA) in 50 mM Tris–HCL, incubate for 30 min at room temperature in the dark. For proteolytic digestion, added 5 μL of 0.1 μg/μL sequencing grade trypsin (Promega, USA) and incubated overnight at 37°C. Concentrated Formic acid (5 μL) was added to stop the reaction and C18 Hypersep plate (bed volume of 40 μL, thermoscientific) was used to cleaned the samples and dried in a vacume concentrator (Eppendorff, Germany).

### 2.5 RPLC‐MS/MS Analysis

To obtain a final concentration of 0.88 μg/μL, samples digest was dissolved in a solvent A and sonicated briefly. Centrifuged at 14000 g for 20 min at 4°C before transferring 12 μL aliquots to sample vials. Solvent A (25 μL) was used for peptides reconstitution and 2.5 μL (ca.2.5ug) samples were injected on a 50 cm long EASy Spray C18 column (ThermoFisher Scientific, USA) connected to anUltiMate 3000 nanoUPLC sytem (Thermofisher Scientific, USA) using a 90 min long gradient: 4%–25% of solvent B (98% CAN). 1% FA in 90 min, 25%–95% in 5 min and 95% of Solvent B for 5 min at flow rate 300nI/min. Mass Spectrometry were acquired Q Exactive HF hybrid quandrupole‐Orbitrap mass spectrometer (ThermoFisher Scientific, USA) ranging from m/z 350 to 16 000 at a resolution of *R* = 120 000 (at m/z 200). It targeted 5 × 10^6^ ions with a maximum injection time of 100 ms, followed by data‐dependent higher‐energy collisional dissociation (HCD) fragmentation of the top 17 precursor ions with charge states ranging from 2+ to 7+, using a 45 s dynamic exclusion. The tandem mass spectra were acquired at a resolution of *R* = 30 000, targeting 2 × 10^5^ ions with a maximum injection time of 54 ms. The quadrupole isolation width was set to 1.4, and the normalized collision energy was adjusted to 28%.

### 2.6 Mass Spectrometry Data Analysis

Against human protein database (SwissProt), acquired raw data file were converted to mascot general (mgf) format using Raw2MGF and searched with Mascot Server v5.1 (MatrixScience, UK). For full tryptic digestion, a maximum of two missed cleaved sites were allowed. The precursor and the fragment ion mass tolerances were set to 10 ppm and 0.02, respectively. However, carbamidomethylation of cysteine was specified as a fixed modification. Deamidation of asparagine, glutamine, and oxidation of methionine were set as dynamic modifications. The initial search was imported to Scaffold, reading the Mascot data files for comparison.

### 2.7 Serum Analysis of PFN‐1 and TAGLN‐2 Levels Using Enzyme‐Linked Immunosorbent Assay (ELISA)

Patients (pre and post NAC) blood (5 mL) was withdrawn by a trained laboratory phlebotomist and was centrifuged, serum was separated and stored at −80°C. Among them, patients with stage I and II were grouped as early‐stage and the patients with stages III and IV were included as advanced stage groups in both pre‐ and post‐NAC patients. Serum PFN‐1 and TAGLN‐2 were measured by ELISA Kit (Bioassay Technology Laboratory, China) according to the manufacturer protocol.

Thirty age‐matched healthy female donors were recruited as controls. Healthy individuals were selected based on the absence of any history of cancer, inflammatory diseases, or other chronic illnesses. The control group was used to establish baseline serum levels of PFN‐1 and TAGLN‐2 and to determine optimal cut‐off values for ELISA analyses.

Briefly, 40 μL serum samples and standards were added in 96 well plates then, the plates were incubated for 24 h at 4°C and each sample was measured in duplicate. After three washes with Phosphate‐Buffered Saline with Tween buffer (PBST), 10 μL primary anti‐PFN‐1 and TAGLN‐2 were added and plates were incubated at room temperature for 1 h. After three washes with buffer PBST, a total of 50 μL streptavidin‐HRP solution was added and mixed well. Cover the plate with a sealer and incubate for 60 min at 37°C. Wash the wells and 50 μL stop solution was added to each well. The absorbance of blank, standard, and samples was measured at 450 nm using a microplate reader (Skanit sky Thermofisher, USA).

### 2.8 RNA Extraction and Reverse Transcription Quantitative Polymerase Chain Reaction (qPCR)

Total RNA was extracted from TNBC patients (pre and post‐NAC) and healthy control whole blood using an extraction kit (Viogene, USA) according to the manufacturer's protocol and immediately stored at −80 C. Using innuSCRIPT One Step RT_PCR SyGreen kit (Analytik Jenax, Germany), isolated RNA (5 ng) was reverse transcribed into cDNA. All primers used in this study are listed in Table 1. In addition, control samples were used as calibrators for relative gene expression analysis in RT‐qPCR using the ΔΔCt method.

**TABLE 1:** Details of primer sequences of the PFN‐1, TAGLN‐2, PTEN, and GAPDH genes used in RT‐qPCR amplification.

Gene name	GENE ID	Forward primer	Reverse primer
Profilins‐1	PFN‐1	CGCCTACATCGACAACCTCATGG	AGCTGGCGTGATGTTGACGAAC
Transglin‐2	TAGLN‐2	AGTGACATTCCCAGAGAGCC	GGCCCCTAAATTTTGGTCCC
Phosphatidylinositol 3,4,5‐trisphosphate 3‐phosphatase	PTEN	CCCACCACAGCTAGAACTTATC	TCGTCCCTTTCCAGCTTTAC
Glyceraldehyde‐3‐phosphate dehydrogenase	GAPDH	ACCCACTCCTCCACCTTTGAC	CTGTTGCTGTAGCCAAATTCG

Briefly, 10 μL of qPCR mixture was used for 1 μL of cDNA and 0.2 μM primers. Each PCR cycle consisted of an initial denaturation at 95°C for 10 s, annealing for 30 s at 60°C and extension at 72°C for 1 min. A total of 35 cycles were used, and each sample was analyzed in triplicate. For the relative quantification, the mean of three experimental values was used. Using the ΔΔCT method [18] relative expression and threshold cycle (CT) of all the target genes (PFN‐1, TAGLN‐2 and PTEN) were evaluated and normalized with GAPDH as a reference gene. Relative expression levels were expressed as ΔCT, which was calculated as ΔCT = CT (target gene) – CT (reference gene) (GAPDH). The 2^ΔΔCT^ values represent the fold change in gene expression and are calculated as ΔCT (target gene)—ΔCT (reference gene) = 2^ΔΔCT^.

### 2.9 In Silico Analysis of the PFN‐1 and TAGLN‐2 by STRING

The search tool STRING version 12.0 database (http://string‐db.org/) was used for protein–protein interaction (PPI) analysis and to explore biological association networks among neighboring partners specifically for TAGLN‐2 and PFN‐1. In the STRING database, the basic interaction unit is the proteins ‘functional association’ that jointly contribute to the same functional process [19].

### 2.10 The Kyoto Encyclopedia of Genes and Genomes (KEGG) Enrichment Analysis

Pathway analysis was performed on the selected proteins and genes PFN‐1 and TAGLN‐2 using online KEGG analysis (https://www.genome.jp/kegg/pathway.html) to examine the enriched terms of biological process [20].

### 2.11 Statistical Analysis

The data was analyzed using SPSS software version 24. Proteins PFN‐1 and TAGLN‐2 levels were compared between pre‐NAC and post‐NAC using the Wilcoxon signed‐rank test, whereas gene expression data were evaluated using the Mann–Whitney U test. The *p*‐value of < 0.05 was considered as statistical significance.

## 3 Results

### 3.1 Mass Spectrometry

To identify the protein features from pre, post‐NAC patients, RPLC‐MS/MS Q Exactive HF hybrid quadrupole‐Orbitrap mass spectrometer qualitative analysis was done. PFN‐1 and TAGLN‐2 were identified in all the samples (Table 2). Each peptide sequence identified for each protein, mass to charge ratio, actual peptide mass, tendency, and retention time are summarized in Table 2. However, spectra of PFN‐1 and TAGLN‐2 peptide sequences are shown in Figures S1 and S2.

**TABLE 2:** PFN‐1 and TAGLN‐2 identified by mass spectrometry.

Protein name	Acc. No	Gene symbol	Mr [kDa]	No of peptide identified	Distinct peptides sequence	m/z	Actual peptide mass (Da)	RT	Tendency
Profilin‐1	P07737	PFN‐1	15	5	(R)SSFYVNGLTLGGQK(C) (K)DSPSVWAAVPGK(T) (K)TFVNITPAEVGVLVGK(D) (R)DSLLQDGEFSMDLR(T) (K)STGGAPTFNVTVTK(T)	735.88 607.31 822.47 813.38 690.36	1469.71 1212.61 1642.93 1624.74 1378.71	4020 3700 5570 5410 2920	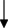
Transgelin‐2	P37802	TAGLN‐2	22	5	(R)TLMNLGGLAVAR(D) (R)EVQQKIEKQYDADLEQILIQWITTQCR(K) (R)ENFQNWLK(D) (K)QMEQISQFLQAAER(Y) (R)DDGLFSGDPNWFPK(K)	608.35 1127.56 539.77 839.92 797.86	1214.68 337 965 1077.53 1677.82 1593.71	4330 4720 3680 4930 5940	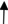

*Note:* Acc. No.: Accession Number; RT: Retention Time; m/z: mass to charge ratio; Mr. KDa: molecular weight in Kilo Dalton; Tendency refers to the trend in the intensities of m/z values between the two groups of TNBC patients; ↑ = higher intensity; ↓ = lower intensity.

### 3.2 Serum PFN‐1 and TAGLN‐2 Levels Relative to Tumor Stages

In an attempt to assess the differential protein expressions of PFN‐1 and TAGLN‐2 in pre‐ and post‐NAC groups, the recruited samples were further stratified into cancer stages (I–IV) and groups as early (stages I and II) and advanced stages (III and IV) in both pre and post‐NAC treated TNBC patients. ELISA with specific anti‐PFN‐1 and TAGLN‐2 antibodies confirmed the levels of two identified proteins in pre and post‐NAC patients.

Healthy individuals were selected based on the absence of any history of cancer, inflammatory diseases, or other chronic illnesses. To discriminate the patient's PFN‐1 and TAGLN‐2 levels with healthy individuals (control), the best cut‐off values 3.01 (2.50–3.89 ng/mL) and 25.57 (16.48–37.65 ng/mL) were achieved for PFN‐1 and TAGLN‐2 respectively from 30 (*n* = 30) aged matched control samples.

### 3.3 PFN‐1 Protein Level in Pre‐NAC and Post‐NAC Group

**FIGURE 2:**
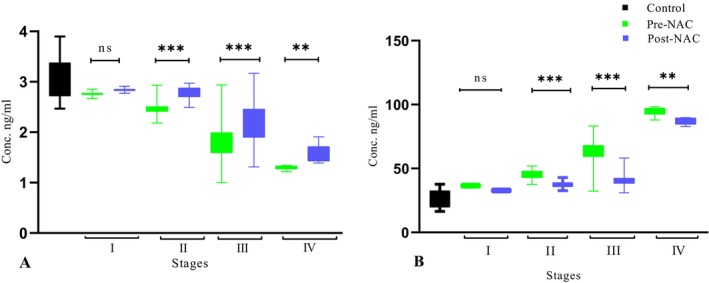
Expression levels of (A) PFN‐1 and (B) TAGLN‐2 in TNBC pre and post‐NAC treated patients. ***p < 0.001; * = *p* < 0.05; ** = *p* < 0.01; ns = not significant.

Serum concentration of PFN‐1 in pre‐NAC was significantly lower than in post‐NAC patients. Figure 2A shows that in the early stages of pre‐NAC, the serum concentration was significantly (*p* = 0.0001) higher than in advanced stages. In the post‐NAC patients with advanced stages, the PFN‐1 level was lower than in early stages.

### 3.4 TAGLN‐2 Protein Level in Pre‐NAC and Post‐NAC Group

The TAGLN‐2 protein concentration was significantly higher (*p* = 0.0001) in the pre‐NAC group compared with the post‐NAC group. However, the TAGLN‐2 level was found to be higher in pre‐NAC advanced stages compared with the post‐NAC group (Figure 2B).

### 3.5 The Differentially Expressed Genes of PFN‐1, TAGLN‐2 and PTEN With Tumor Stages

House‐keeping GAPDH gene was used for normalisation the ΔCT and ΔΔCT values by using the following formulas:

$$\mathrm{CT}\;\left(\text{target gene}\right)\_\mathrm{CT}\;\left(\text{Reference gene}\right)=\mathrm{\Delta CT}$$

$$\mathrm{\Delta CT}\;\left(\text{Sample}\right)\_\mathrm{\Delta CT}\;\left(\text{Calibrator}\right)=\text{ΔΔCT}$$

$$\text{Relative fold change in expression}=2^{\text{ΔΔCT}}$$

The relative fold change in the mRNA expression level of all the genes between the pre and post NAC was shown as the 2^ΔΔCT^ (Tables 3 and 4) and a *p* value of < 0.05 is considered statistically significant. Remarkably, the combination of three genes is differentially expressed among all the groups and their tumor stages.

**TABLE 3:** ΔCT mean, ΔΔCT, 2^ΔΔCT^ and *p* values for differentially expressed genes of PFN‐1, TGLN‐2 and PTEN in Pre‐ NAC TNBC patients.

Genes	Stages	ΔCT (patients)	ΔCT (control)	ΔΔCT	2^−ΔΔCT^	95% confidence interval	*p*
PFN‐1	I & II	5.926	5.500	0.428	0.755	0.547–0.964	0.093
	III & IV	6.692	5.500	1.194	0.524	−0.331–0.717	
TGLN‐2	I & II	2.264	3.870	−1.523	1.301	2.103–3.933	0.322
	III & IV	1.982	3.870	−1.806	3.605	0.385–4.453	
PTEN	I & II	6.010	4.260	1.748	0.303	−1.101–0.081	0.750
	III & IV	6.121	4.560	1.859	0.347	−0.142–0.553	

**TABLE 4:** ΔCT mean, ΔΔCT, 2−ΔΔCT and *p* values for differentially expressed genes of PFN‐1, TGLN‐2 and PTEN in Post NAC TNBC patients.

Genes	Stages	ΔCT (patients)	ΔCT (control)	ΔΔCT	2^−ΔΔCT^	95% confidence interval	*p*
PFN‐1	I & II	6.764	5.690	1.267	0.756	0.350–1.229	0.965
	III & IV	6.210	5.100	0.713	0.211	0.516–1.017	
TGLN‐2	I & II	7.496	3.870	3.623	0.160	0.068–0.312	0.002*
	III & IV	3.812	3.870	0.025	2.015	1.233–2.931	
PTEN	I & II	8.383	4.260	4.714	0.130	0.0337–0.3007	0.011*
	III & IV	5.000	4.260	0.647	1.036	0.6363–1.5939	

*Note:* Significant * = *p* < 0.05.

#### 3.5.1 PFN‐1 Gene Expression in Pre‐NAC and Post‐NAC Groups

**FIGURE 3:**
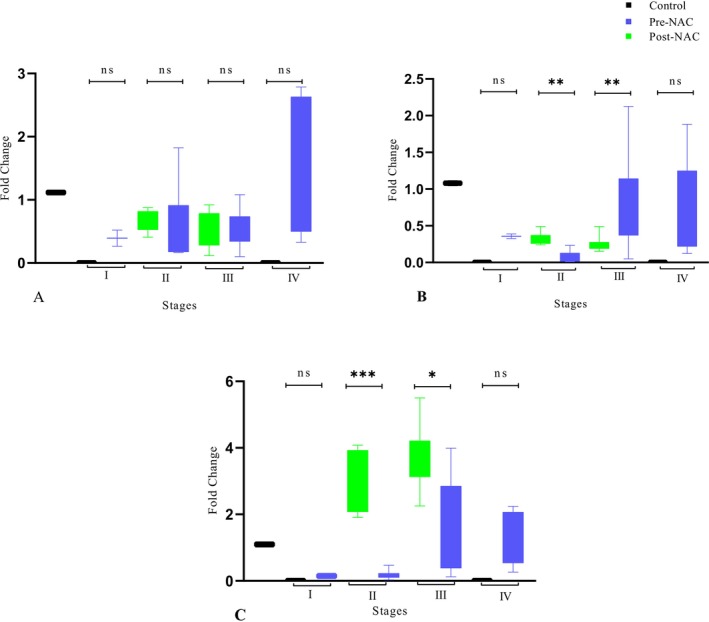
Showing relative fold change of (A) PFN‐1, (B) PTEN (tumor suppressor gene) and (C) TAGLN‐2 expressions in pre and post‐NAC treated TNBC patients with different stages validated by qPCR. Housekeeping gene GAPDH mRNA expression was used for data normalization. The *p*‐value < 0.005 was considered as statistically significant after three individual experiments and error bars indicate ± SD. ***p < 0.001; * = *p* < 0.05; ** = *p* < 0.01; ns = not significant.

In the pre‐NAC group, the PFN‐1 gene was upregulated at early stages (I & II) compared with advanced stages (III & IV). However, post NAC was shown to be significantly downregulated of the PFN‐1 gene at advanced stages (Figure 3A).

#### 3.5.2 TAGLN‐2 Gene Expression in Pre‐NAC and Post‐NAC Groups

Interestingly, gene TAGLN‐2 is highly overexpressed in the pre‐NAC group at advanced stages; however, the post NAC group was also shown to have overexpression of TAGNL‐2 genes at advanced stages compared with early stages of tumors (Figure 3C).

#### 3.5.3 PTEN Gene Expression in Pre‐NAC and Post‐NAC Groups

PTEN (phosphatase and tensin homolog) is a known tumor suppressor gene that negatively regulates the PI3K/AKT signaling pathway, which is frequently hyperactivated in TNBC. Its loss leads to uncontrolled cell proliferation, survival, and metastasis. In light of its significance, we assessed PTEN gene expression across all the study groups. In the current study, tumor suppressor gene PTEN was downregulated at early stages of pre and post‐NAC groups and upregulated at advanced stages of post‐NAC stages (Figure 3B).

### 3.6 STRING Analysis

**FIGURE 4:**
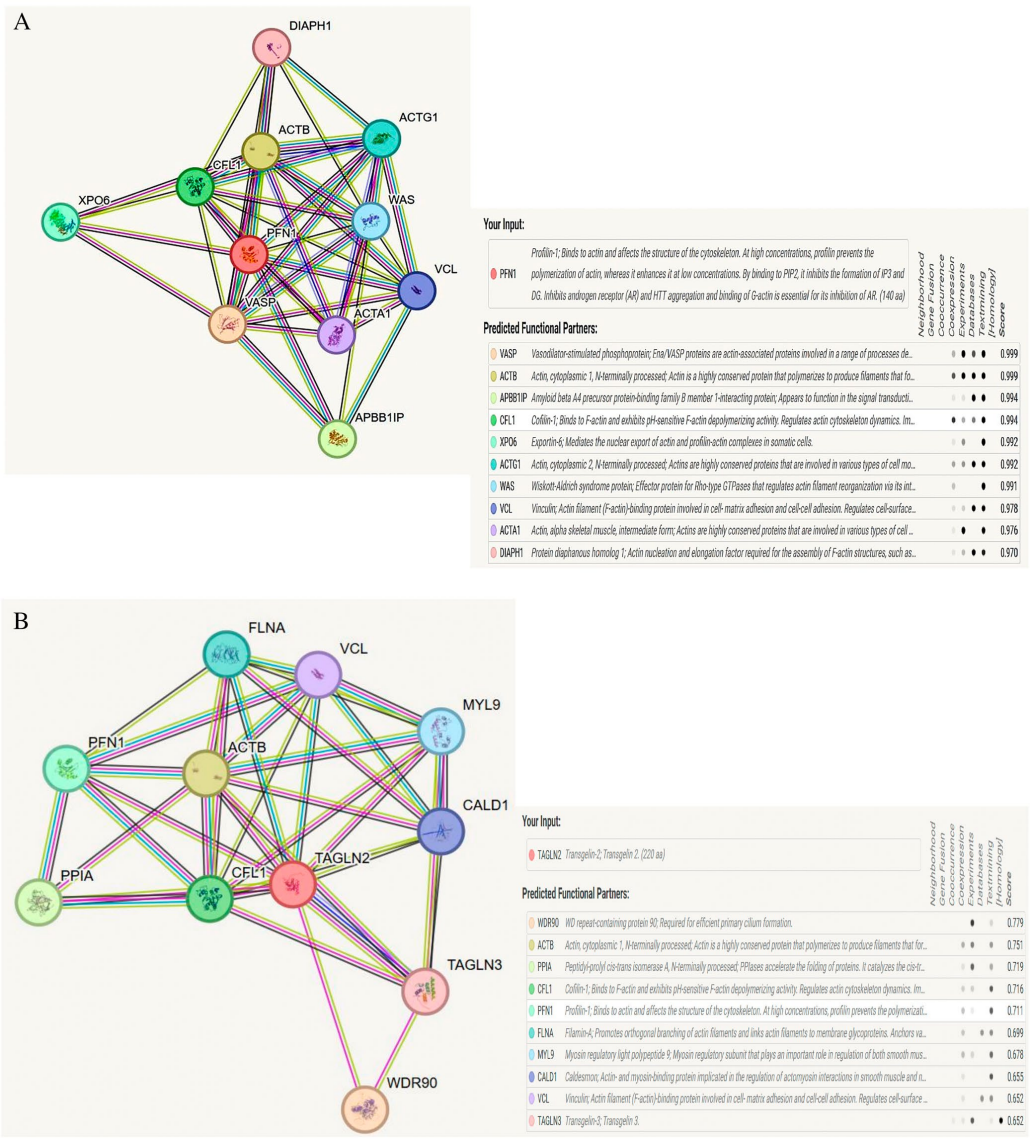
Interactome neighborhood of PFN‐1 (A) and TAGLN‐2 (B) among homo‐sapiens as retrieved by STRING database. Using STRINGv12.0 analysis tools (http://string‐db.org), confidence view of a protein–protein interactions map was generated. PFN‐1 (A) predicted functional partners were VAMP and Actin with confidence score 0.999. TAGLN‐2 (B) predicted functional partners were Actin and PFN‐1 with confidence score 0.751 and 0.711 respectively. PFN‐1and TAGLN‐2 are highlighted in Red.

Among homo‐sapiens, the interactome neighborhood of PFN‐1 and TAGLN‐2 were retrieved and analyzed by a computational tool, STRING database. As shown in Figure 4A,B, PFN‐1 and TAGLN‐2 both showed 10 reputed interactors, however, PFN‐1 was showed high confidence score of 0.999 with vasodilator‐stimulated phosphoprotein (VAMP) and actin proteins which confirms its maximum interaction. TAGLN‐1 showed its maximum interaction with WD repeat‐containing protein 90 (WDR90), actin, peptidyl‐prolyl cis‐trans isomerase A (PPIA), cofilin I (CFL‐1) and PFN‐1 with high confidence score 0.779, 0.751, 0.719, 0.716 and 0.711 respectively.

### 3.7 The Kyoto Encyclopedia of Genes and Genomes (KEGG) Enrichment Analysis

**FIGURE 5:**
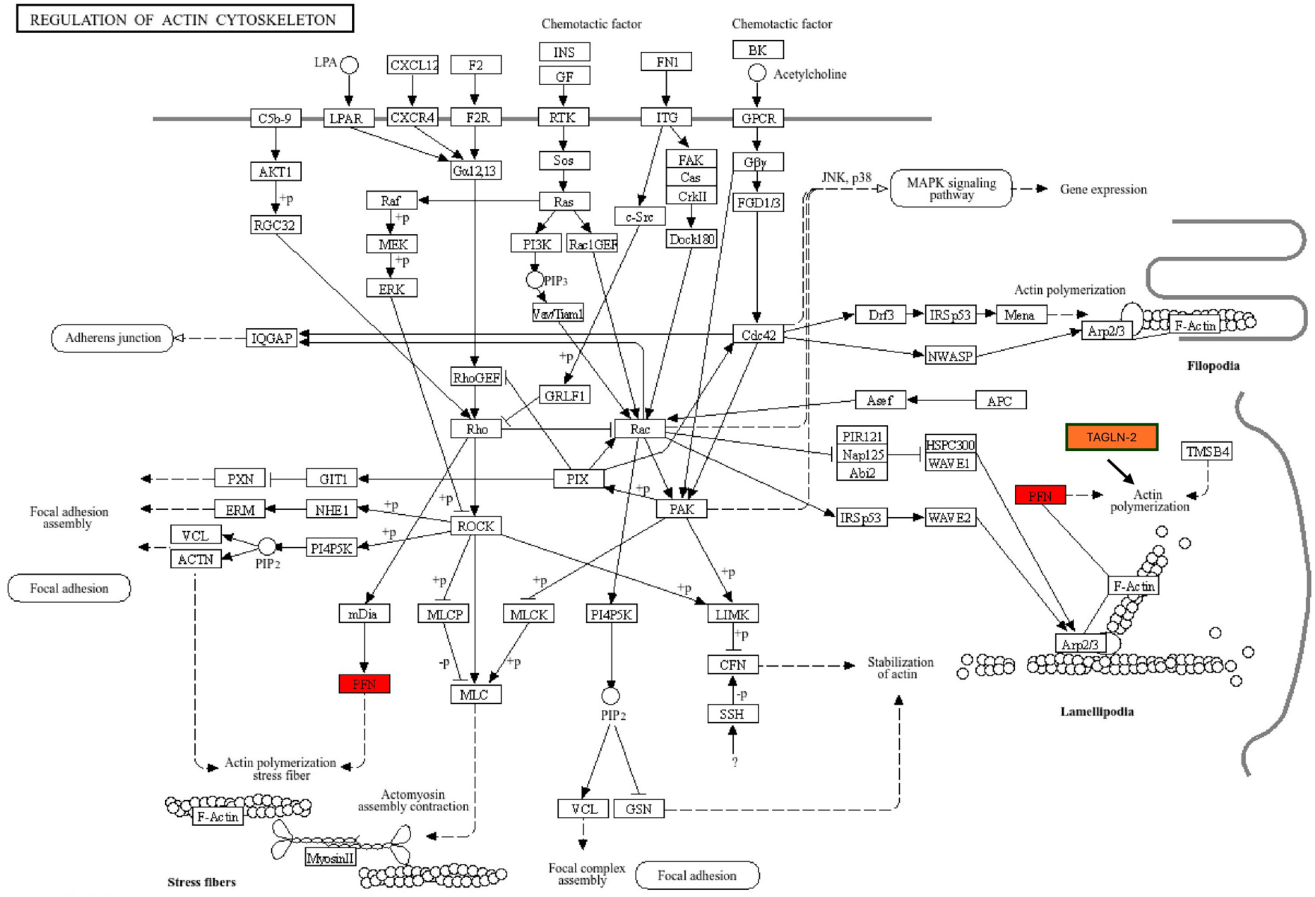
KEGG pathway analysis of PFN‐1 and TAGLN‐2 enriched in regulation of actin cytoskeleton. TGNLN‐2 is also involved in actin polymerization therefore added additionally and highlighted with orange color.

This database is widely used for several aspects such as cellular processes, human diseases, metabolism, drug development, environmental and genetic information processing for differential expressed proteins [20]. The identified proteins PFN‐1 and TAGLN‐2 pathway analysis was carried out using KEGG pathway enrichment analysis displayed association with regulation of actin cytoskeleton (Figure 5) which may provide insights and a theoretical basis for further understanding the pathogenesis of actin‐binding proteins in TNBC.

## 4 Discussion

Microfilament‐associated proteins identified in blood cells could significantly aid in the development of non‐invasive approaches for early and predictive breast cancer diagnosis. Previous studies have hypothesized that poor cytoskeletal architecture contributes to neoplastic transformation.

Microfilament members are functionally interconnected and play a crucial role in all stages of tumorigenesis, from precancerous lesions to the enhanced metastatic potential of malignancies. In particular, the loss of actin or poorly arranged actin‐skeleton organizations is associated with increasing metastatic potential of tumor cells [21].

Cells need to tightly control their motility for transitions between epithelial and mesenchymal states and control occurs at multiple levels, including reorganization of the actin cytoskeleton, gene expression and post‐translational modifications. Recently, it has been reported that the reorganization of the actin cytoskeleton during the epithelial‐mesenchymal transition (EMT) is regulated by various actin‐binding proteins (ABPs), which play a key role in tumor cell progression [22, 23].

PFN‐1 and TAGLN‐2 are being implicated for the first time as prognostic biomarkers in TNBC, with their gene expression changes being significant in predicting patient outcomes across different stages of pre and post‐NAC groups. PFN‐1 is associated with tumor progression, but the exact mechanisms behind its altered expression remain unclear. Various studies have highlighted its potential role in cancer biology [24, 25, 26] but why its expression is altered in TNBC patients specifically is still not fully understood.

It has been reported that reduced PFN‐1 expression correlates with increased metastatic activity in breast cancer in female nude mice [12]. Conversely, an in vivo study has shown that PFN‐1 modulates breast cancer aggressiveness, and its genetic overexpression reduces tumorigenesis, cell migration, and invasion in breast cancer [26].

We found a higher expression of serum PFN‐1 level in patients at early stages of pre‐NAC compared with advanced stages; however, reduced protein levels of PFN‐1 were present at advanced stages in post‐NAC, which could be NAC response. Similarly, in the pre‐NAC group, the PFN‐1 gene was upregulated at early stages compared with advanced stages, and reduced mRNA expression in the post‐NAC group at advanced stages was observed. It was known that mRNA levels were not always correlated with protein levels and their activation. A study reported that PFN‐1 is a cogent tissue biomarker of clinical prognosis but also indicates a potential causality between PFN‐1 dysregulation and tumor progression in renal cancer [27]. Previously it was well established that PFN‐1 interacts with Mena/VASP protein (regulator of actin cytoskeletal structure) to regulate actin polymerization to promote cell migration [28].

**FIGURE 6:**
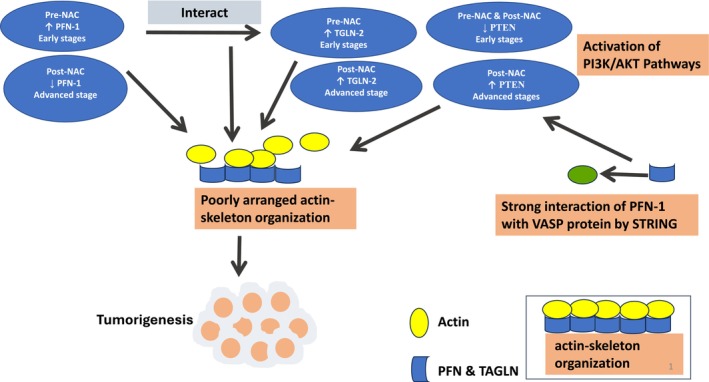
Mechanistic Model is Showing Aberrantly Expressions of PFN‐1, TAGLN‐2 and PTEN Markers in Tumor Stages of TNBC Patients. Receiving Neoadjuvant Chemotherapy.

Interestingly, a current bioinformatic study (STRING) has also shown a strong interaction of PFN‐1 with VASP protein. We have shown the involvement of PFN‐1 in TNBC not only by its level and interaction with other regulators but also by its relationship with other identified proteins (Figure 6 Mechanistic Model).

In the current study, PFN‐1 and TAGLN‐2 pathway analysis was carried out using KEGG pathway enrichment analysis displayed association with regulation of actin cytoskeleton (Figure 5). Therefore, we speculate that PFN‐1 promotes disease progression not only through its actin interaction but with TAGLN‐2 and VASP as well. This finding is based on bioinformatics data and lacks direct experimental validation. While bioinformatics methods are valuable in generating hypotheses, further investigation validation is essential to confirm the findings.

Current finding was also revealed that gene TAGLN‐2 is highly overexpressed in the pre‐NAC group at advanced stages; however, the post NAC group has shown overexpression of TAGLN‐2 genes at advanced stages compared with early stages of tumors. Moreover, a high level of TAGLN‐2 may activate the PI3K/Akt/GSK‐3b pathway by interacting with PTEN, thereby contributing to the Paclitaxel (first line chemotherapy for breast cancer) resistance, as well as promoting cell migration and invasion of breast cancer (Figure 6 Mechanistic Model). Liu Yang et al. showed TAGLN‐2 expression is associated with lymph node and distant metastasis, both of which are key prognostic factors in breast cancer patients [17, 29]. They have also revealed that TAGLN‐2 as a tumor suppressor may promote the metastasis of breast cancer by activating the nuclear factor‐κB signaling pathway.

Zaidi AH [30] and his co‐workers established that PFN‐1 interacts with PTEN (phosphatase and tensin homolog), a phosphatase, and stabilizes it. When PTEN is suppressed or mutated, the PI3K/AKT pathway becomes hyperactivated, resulting in uncontrolled cell proliferation, evasion, and enhanced survival. This loss of regulation is a key factor in the development and progression of breast cancer, contributing to both tumorigenesis and metastasis. Given the significance of the PTEN gene in cancer initiation and prognosis, the present study also aims to evaluate the PTEN gene dysregulation in our TNBC pre and post NAC patients. Despite extensive global research on the implications of the PTEN gene in breast cancer, the precise mechanism of altered PTEN involvement with other markers is not well established.

A north Indian study on breast cancer showed that PTEN genetic and epigenetic alteration is associated with clinical stage of breast cancer [31] however, deletion of PTEN contributes to the occurrence and development of gastric cancer [32]. A targeted exome sequencing of 41 metastatic breast cancer tumors, 10% was found PTEN mutations [33]. Current study revealed that PTEN gene was down regulated in early stages of pre‐NAC and post‐NAC patients. Previous studies have reported PTEN loss in breast cancer progression [34, 35], with its loss being associated with larger tumor size [36]. An in vitro study reported PTEN over expressed MCF‐7 cells showed decreased chemo resistance that showed tumor suppressive effect of PTEN which is crucial for cancer prevention [37].

Studies have shown that PTEN acts as a negative regulator of phosphoinositide 3‐kinase (PI3K) activation by dephosphorylating phosphatidylinositol‐3,4,5‐triphosphate (PIP3) into phosphatidylinositol‐4,5‐bisphosphate (PIP2).

During breast cancer development, PTEN inactivation leads to PI3K pathway hyperactivation, inhibition of apoptosis, uncontrolled cell proliferation, and tumor progression [38, 39].

The loss of PTEN and elevated PI3K expression are associated with poor outcomes in TNBC patients and play a significant role in TNBC progression [40].

These findings highlight the clinical significance of PTEN in breast cancer biology and its potential role as a therapeutic target (Figure 6 Mechanistic Model). Contrary, we did not show a significant connection of tumor suppressor gene PTEN with PFN‐1 and TAGLN‐2 expression; therefore, we cannot confirm that the suppressed expression of PTEN at early stages can promote the PFN‐1 or TAGLN‐2 gene expression in TNBC pre‐ and post NAC patients.

Further research is needed to elucidate the underlying mechanisms driving the associations of PTEN with PFN‐1 and TAGLN‐2 and explore the potential therapeutic implications of targeting the PTEN pathway in TNBC management.

This study includes PTEN gene expression; however, the limited sample size and lack of a strong direct correlation between PTEN and the PFN‐1 and TAGLN‐2 markers limit the ability to confirm mechanistic links.

## 5 Conclusion

This study underscores the potential of actin‐binding proteins PFN‐1 and TAGLN‐2 as non‐invasive biomarkers for breast cancer, particularly TNBC. Serum levels of PFN‐1 were higher in early‐stage patients and decreased in advanced stages, especially in post‐NAC, implying a potential role in treatment response. However, TAGLN‐2 expression was elevated at advanced stages in the pre‐NAC group compared with post‐NAC, supporting its involvement in tumor aggressiveness. These protein expressions in serum may reflect tumor‐specific processes rather than systemic inflammation, providing a promising avenue for non‐invasive early detection and monitoring strategies. The observed interactions of PFN‐1 with VASP and potential crosstalk with TAGLN‐2 point toward their collective role in actin cytoskeleton regulation and tumorigenesis. While PTEN dysregulation was confirmed in TNBC patients, no direct correlation with PFN‐1 or TAGLN‐2 expression was established. Before effective targeted cancer therapies can be developed, a deeper understanding of the molecular pathways involving actin‐binding proteins and PTEN in cancer progression is required, supported by studies with larger sample sizes.

## Author Contributions

Conception/design: S.M. won a research grant from the Higher Education Commission (HEC), Karachi Pakistan, Provision of study material or patients: G.H. diagnosed cases and provided TNBC blood samples and supported in assembly of data, M.A.S.; Performed the experiments, A.K. performed KEGG analysis, M.A.S., U.N. and S.M.; Data analysis and interpretation. S.M.; Manuscript writing: B.W.; manuscript review, all authors approved the final draft of the manuscript.

## Funding

This research was funded by Higher Education Commission (HEC), Karachi Pakistan grant (Reference no. 20‐11952/NRPU/RGM/R&D/HEC).

## Ethics Statement

This study conforms to the ethical guidelines of the Declaration of Helsinki. The study protocol was reviewed and approved by the Research Ethics Committee (ERC) of Ziauddin University (Reference code: 1730120SMBIO).

## Consent

Informed consent was obtained from all the participants and their legal guardians.

## Conflicts of Interest

The authors declare no conflicts of interest.

## Supporting information


**Figure S1:** Representative MS/MS spectra of peptides derived from PFN‐1 (Profilin‐1) identified by mass spectrometry. The annotated b and y‐ion fragment series confirm the peptide sequences used for PFN‐1 protein identification.

**Figure S2:** Representative MS/MS spectra of peptides derived from TAGLN‐2 (Transgelin‐2) identified by mass spectrometry. The annotated b and y‐ion fragmentation patterns confirm the peptide sequences used for TAGLN‐2 protein identification.

**Table S1:** Baseline characteristics of TNBC patients (*n* = 54).

## Data Availability

The data that supports the findings of this study are available in the Supporting Information of this article.
