# Supplementary figures and images for: Profillin‐1 and Transgelin‐2: Actin Binding Proteins Expression in Early and Advanced Stages of Triple‐Negative Breast Cancer Receiving Neoadjuvant Chemotherapy
Source: Cancer Rep (Hoboken). 2026 Mar 30;9(4):e70529. doi: 10.1002/cnr2.70529 (PMC13140608; doi:10.1002/cnr2.70529)

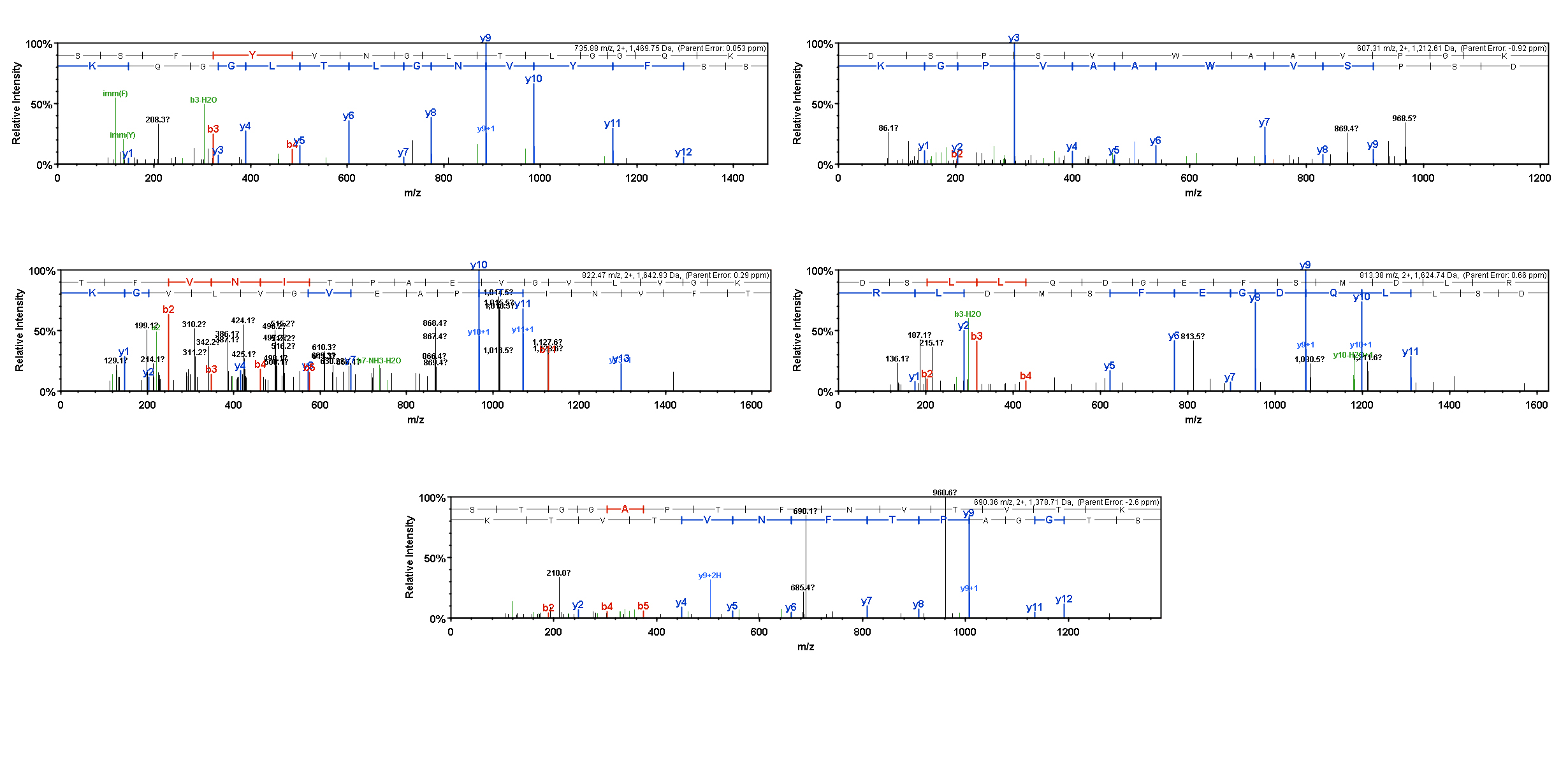

Supplement: Supplementary file 1 — Figure S1: Representative MS/MS spectra of peptides derived from PFN‐1 (Profilin‐1) identified by mass spectrometry. The annotated b and y‐ion fragment series confirm the peptide sequences used for PFN‐1 protein identification. [file CNR2-9-e70529-s001.jpg]

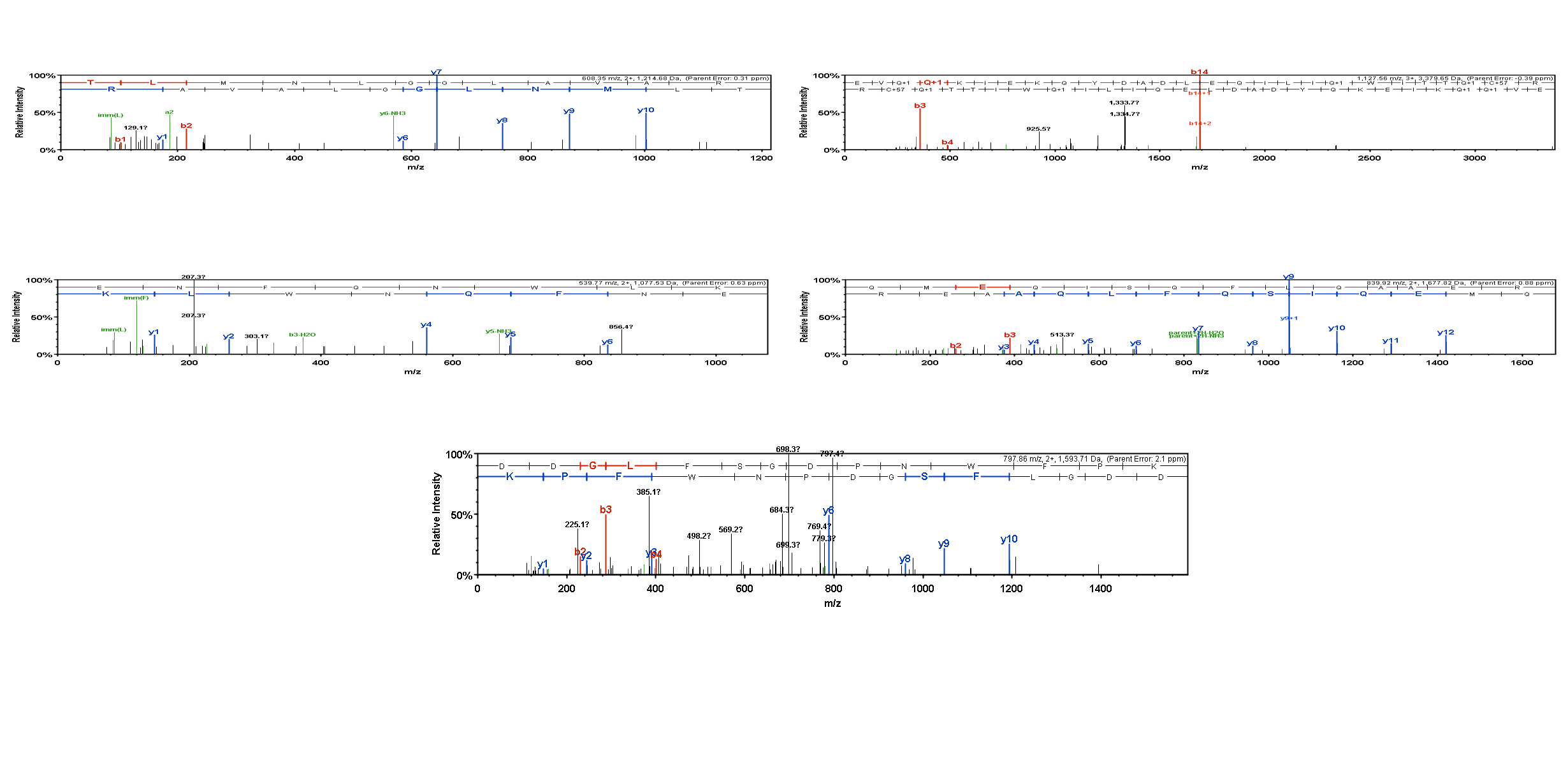

Supplement: Supplementary file 2 — Figure S2: Representative MS/MS spectra of peptides derived from TAGLN‐2 (Transgelin‐2) identified by mass spectrometry. The annotated b and y‐ion fragmentation patterns confirm the peptide sequences used for TAGLN‐2 protein identification. [file CNR2-9-e70529-s002.jpg]
