# Supplementary material for: Profillin‐1 and Transgelin‐2: Actin Binding Proteins Expression in Early and Advanced Stages of Triple‐Negative Breast Cancer Receiving Neoadjuvant Chemotherapy
Source: Cancer Rep (Hoboken). 2026 Mar 30;9(4):e70529. doi: 10.1002/cnr2.70529 (PMC13140608; doi:10.1002/cnr2.70529)
Supplement: Supplementary file 3 — Table S1: Baseline characteristics of TNBC patients (n = 54). [file CNR2-9-e70529-s003.docx]

| **Average age (Years)** |  |
| --- | --- |
| 24- 45 **Mean ± standard deviation 37.5 ± 5.1 years.** | |
| **T Stage n(%)** | |
| cT1 | 1(1.86) |
| cT2 | 18(33.33) |
| cT3 | 29(53.70) |
| cT4 | 6(11.11) |
| **N Stage** | |
| cN0 | 5(9.25) |
| cN1 | 43(79.6) |
| cN2 | 4(7.40) |
| cN3 | 2(3.7) |
| **M Stage** | |
| M0 | 51(94.44) |
| M1 | 3(5.55) |
| **Tumor subtype** | |
| **IDC** | 54(100) |
| **Histological grade** | |
| I | 1(1.85) |
| II | 11(20.37) |
| III | 42(77.77) |
| **Surgery** | |
| MRM | 50(92.59) |
| BCS | 4(7.40) |
| **Family History** | |
| Yes | 4(7.40) |
| No | 50(92.59) |

**Supplementary table 1: Baseline characteristics of TNBC patients (n=54)**
